# Sex‐Specific Differences in the Predictive Value of Cholesterol Homeostasis Markers and 10‐Year Cardiovascular Disease Event Rate in Framingham Offspring Study Participants

**DOI:** 10.1161/JAHA.112.005066

**Published:** 2013-02-22

**Authors:** Nirupa R. Matthan, Lei Zhu, Michael Pencina, Ralph B. D'Agostino, Ernst J. Schaefer, Alice H. Lichtenstein

**Affiliations:** 1 Cardiovascular Nutrition Laboratory, Jean Mayer USDA Human Nutrition Research Center on Aging, Tufts University, Boston, MA (N.R.M., A.H.L.); 2 Lipid Metabolism Laboratory, Jean Mayer USDA Human Nutrition Research Center on Aging, Tufts University, Boston, MA (E.J.S.); 3 Department of Biostatistics, Boston University, Boston, MA (L.Z., M.P., R.B.A.)

**Keywords:** cardiovascular disease, lipids, metabolism, mortality, myocardial infarction, risk factors

## Abstract

**Background:**

Available data are inconsistent regarding factors influencing plasma cholesterol homeostasis marker concentrations and their value in predicting subsequent cardiovascular disease (CVD) events.

**Methods and Results:**

To address this issue, the relationship between markers of cholesterol absorption (campesterol, sitosterol, cholestanol) and synthesis (squalene, desmosterol, lathosterol) and 10‐year CVD incidence was assessed in Framingham Offspring Study participants (cycle 6) who were without CVD at baseline and not taking lipid‐lowering medications (N=2616). The primary end point was “hard” coronary heart disease (HCHD; coronary death and myocardial infarction), and the secondary end point was full CVD (HCHD plus stroke, coronary insufficiency, angina pectoris, peripheral artery disease, and congestive heart failure). In cross‐sectional analysis, significant differences by sex, age, body mass index, blood pressure, and smoking status were observed. In both women and men, lower cholesterol absorption was associated with higher triglyceride and lower high‐density lipoprotein (HDL) cholesterol concentrations, whereas lower cholesterol synthesis was associated with higher low‐density lipoprotein (LDL) cholesterol concentrations (*P* for trend <0.05). In women only, lower cholesterol synthesis and absorption were associated with higher non–HDL cholesterol concentrations. Using Cox proportional hazards model adjusting for standard CVD risk factors, squalene concentrations were associated with lower HCHD in women (hazard ratio=0.70 [0.5 to 0.9]). In contrast, squalene (hazard ratio=1.40 [1.1 to 1.8]) concentrations were associated with higher HCHD in men (*P*<0.0001 for interaction). The cholesterol absorption markers were not predictive of HCHD or full CVD in either women or men.

**Conclusions:**

These data suggest significant sex differences in the 10‐year prognostic value of cholesterol synthesis markers and HCHD, specifically coronary death and incidence of myocardial infarction.

**Clinical Trial Registration:**

URL:http://ClinicalTrials.gov. Unique identifier: NCT00074464.

## Introduction

High low‐density lipoprotein cholesterol (LDL‐C) and triglyceride concentrations and low high‐density lipoprotein cholesterol (HDL‐C) concentrations are established risk factors for cardiovascular disease (CVD).^1^ Cholesterol concentrations within the circulatory pool are products of input from gut absorption and endogenous synthesis relative to clearance through hepatic and extrahepatic tissue pathways.^2^ A disruption in any of these mechanisms can alter this balance, which is reflected in plasma cholesterol concentrations and subsequent CVD progression.^3–4^ The importance of cholesterol synthesis as a regulator of plasma LDL‐C concentrations has been demonstrated through studies reporting an inverse relationship between 3‐hydroxy‐3‐methylglutaryl–coenzyme A (HMG‐CoA) reductase inhibitors (statin therapy) and cholesterol synthesis rates,^5–10^ and dietary cholesterol and de novo biosynthesis rates.^11^ Similarly, studies have demonstrated that altering the efficiency of cholesterol absorption plays a critical role in determining LDL‐C and HDL‐C concentrations.^12–13^

Using circulating indicators of cholesterol homeostasis—plasma phytosterol and cholestanol concentrations, which are validated markers of cholesterol absorption, and squalene, desmosterol, and lathosterol concentrations, which are validated markers of cholesterol synthesis^13–15^—we have previously documented in patients with coronary artery disease that LDL‐C concentrations were positively associated with cholesterol synthesis markers and negatively associated with cholesterol absorption markers.^16^ In contrast, HDL‐C concentrations were negatively associated with cholesterol synthesis markers and positively associated with absorption markers. Interestingly, a positive association between HDL‐C concentrations and cholesterol absorption efficiency has been reported in obese and hypercholesterolemic individuals without heart disease.^17^

Given this association between cholesterol homeostasis and plasma lipoprotein concentrations, we and others have started assessing whether cholesterol absorption and synthesis are also associated with prevalent CVD. Results from these cross‐sectional case–control studies have been equivocal with some studies reporting that higher cholesterol absorption and/or lower cholesterol synthesis marker concentrations are associated with increased,^18–25^ decreased,^26–27^ or no difference in^28–30^ CVD risk. Of note, the cohorts used in these studies differed in baseline characteristics, including mean age, body mass index (BMI), and plasma lipoprotein profile, all of which could affect the stage of the disease and thus the magnitude or direction of the associations observed. Also, limits in sample sizes and study duration have potentially precluded assessment of the prognostic value of the cholesterol homeostasis markers on CVD events. The overall objective of the present study was to determine prospectively the validity of cholesterol absorption and synthesis markers as predictors of CVD events during a 10‐year period in Framingham Offspring Study (FOS) participants without prevalent CVD at baseline and not taking lipid‐lowering medication. A secondary objective was to assess cross‐sectionally the relationship between markers of cholesterol absorption/synthesis and established CVD risk factors.

## Methods

### Study Population

The FOS is a longitudinal community‐based study initiated in 1971 with a sample of 5135 men and women, consisting of the offspring of the original Framingham Heart Study cohort^31^ and their spouses. Of the 3532 participants who underwent a standardized medical history and physical examination at the sixth examination cycle (1995–1998), we identified 1785 women and 1593 men without CVD (<5% carotid stenosis and no myocardial infarction, coronary insufficiency, angina pectoris, peripheral artery disease, heart failure, or stroke before the sixth examination cycle) who were not taking lipid‐lowering medications (statins, cholestyramine, niacin, or fibrates). Plasma samples for measurement of cholesterol homeostasis markers were available for 1463 women and 1153 men. The baseline characteristics of subjects with and without cholesterol homeostasis data were similar. This study was approved by the institutional review boards for human research at Tufts University–Tufts Medical Center and Boston University.

### CVD Outcomes

“Hard” coronary heart disease (HCHD) was defined as a composite of coronary death and myocardial infarction. Full CVD was defined as HCHD plus stroke (atherothrombotic infarction, hemorrhagic stroke, and transient ischemic attack), coronary insufficiency and angina pectoris, peripheral artery disease (intermittent claudication), and congestive heart failure.^32^ HCHD and full CVD events were confirmed by medical histories, physical examinations at the study clinic, hospitalization records, and communication with personal physicians as previously described.^3,31^

### Anthropometric and Biochemical Measures

Height, weight, and waist circumference were measured with the subject standing. BMI was calculated (kg/m^2^). Subjects were classified as hypertensive if their diastolic blood pressure was ≥85 mm Hg or systolic blood pressure was ≥130 mm Hg or if use of antihypertensive medications was reported. Current smokers were defined as those who reported smoking ≥1 cigarette per day during the previous year. Estrogen use was classified as current or no use at the time of the examination. Diabetes was defined as fasting glucose ≥126 mg/dL or use of insulin or oral hypoglycemic medications. Metabolic syndrome (MetS) was defined as having ≥3 of the following individual components: abdominal obesity (for men, waist circumference ≥102 cm; for women, waist circumference ≥88 cm), low HDL‐C (for men, <40 mg/dL; for women, <50 mg/dL), elevated blood pressure (≥130/85 mm Hg) or treatment of hypertension, elevated glucose (≥100 mg/dL) or treatment of hyperglycemia, and elevated triglyceride levels (≥150 mg/dL) or treatment for hypertriglyceridemia.

Fasting plasma total cholesterol, HDL‐C, triglyceride, and glucose concentrations were measured using standard enzymatic methods, as previously described.^33–34^ LDL‐C concentrations were calculated according to the formula of Friedewald et al.^35^ The assays were standardized through the Lipid Standardization Program of the Centers for Disease Control and Prevention (Atlanta, GA).

### Cholesterol Homeostasis Analyses

Plasma concentrations of the cholesterol absorption and synthesis markers were quantified using gas chromatography as previously described.^16,36^ Peaks of interest were identified by comparison with authentic standards (Supelco, Bellefonte, PA) and expressed relative to the internal standard. Interassay coefficient of variation was on average ≤5.5% for most of the sterols. External quality control samples were routinely interspersed per 20 samples and analyzed with study samples. The cholesterol homeostasis markers measured included the cholesterol synthesis precursors (squalene, desmosterol, and lathosterol) and the absorption markers (cholestanol, campesterol, and sitosterol). Their concentrations have been expressed relative to the concentration of plasma total cholesterol (μmol/mmol of cholesterol) to correct for the different number of lipoprotein acceptor particles.

### Statistical Analyses

All variables were summarized using means and SEM. Two‐sample *t* test was used to compare cholesterol homeostasis markers between women and men. ANOVA was used to determine the equality of mean cholesterol homeostasis markers among different categories. Sex‐specific Cox proportional hazards models were used to relate the cholesterol homeostasis markers to the incidence of a first CVD event during a maximum follow‐up period of 10 years after confirming that the assumption of proportionality of hazards was met. We focused on HCHD as the primary outcome and full CVD as a secondary outcome. Covariates included in the Cox models were age, BMI, blood pressure, antihypertensive medication, LDL‐C, HDL‐C, triglycerides, smoking, and diabetes status. Estrogen use was not included because there was no statistical difference in the cholesterol homeostasis markers between premenopausal and postmenopausal women. Triglyceride concentrations and all cholesterol homeostasis markers were log‐transformed in the models to correct for their skewed distributions and standardized to express the results on a comparable scale. All analyses were performed using SAS version 9.2 or higher, and *P* values <0.05 were considered statistically significant.

## Results

### Cholesterol Homeostasis Markers and CVD Outcomes

**Figure 1.**
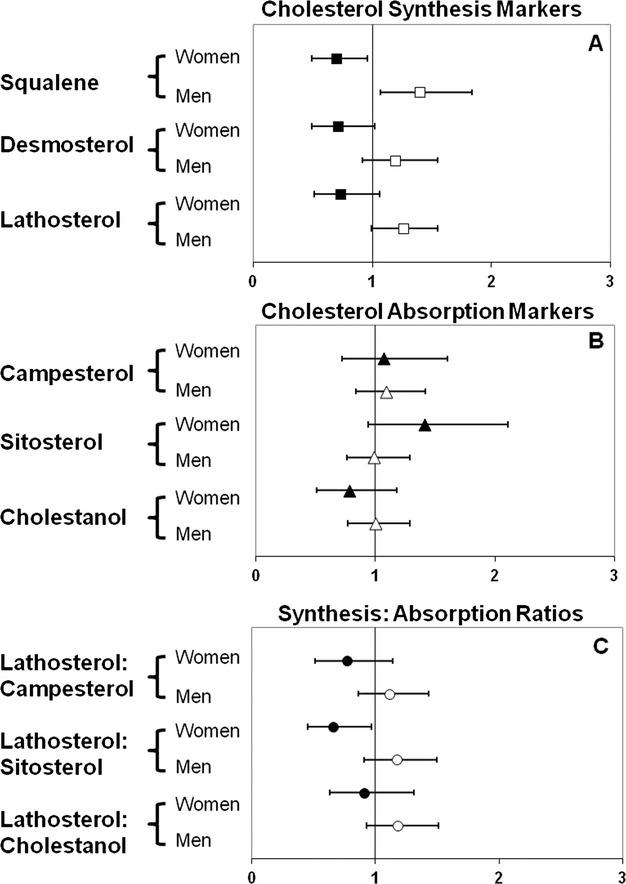
Multivariable‐adjusted hazard ratios for cholesterol synthesis markers (A), cholesterol absorption markers (B), cholesterol synthesis:absorption ratios (C), and “hard” coronary heart disease (coronary death and myocardial infarction) based on Cox proportional hazards model. Error bars represent 95% CIs.

**Figure 2.**
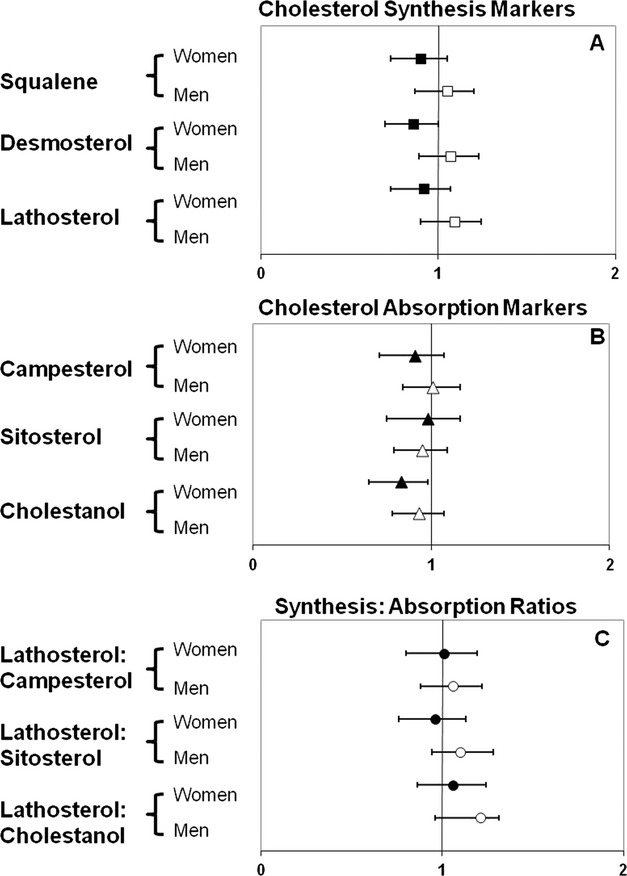
Multivariable‐adjusted hazard ratios for cholesterol synthesis markers (A), cholesterol absorption markers (B), cholesterol synthesis:absorption ratios (C), and full cardiovascular disease (“hard” coronary heart disease plus stroke, coronary insufficiency, angina pectoris, peripheral artery disease, and congestive heart failure) based on Cox proportional hazards model. Error bars represent 95% CIs.

There was a marked difference in the relationship of cholesterol synthesis markers and HCHD risk between women and men. Using Cox proportional hazards models, after controlling for standard risk factors, diabetes, and antihypertensive medication use, squalene was associated with a lower risk of HCHD (hazard ratio [HR]=0.70 [0.5 to 0.9] in women but with a higher risk of HCHD in men (HR=1.40 [1.1 to 1.8]). Values are HR per 1 SD of log with 95% CIs. The *P* value for interaction between men and women was significant (*P*<0.0001). These sex‐specific differences were also observed with desmosterol (HR=0.71 [0.5 to 1.0] and HR=1.19 [0.9 to 1.5] for women and men, respectively) and lathosterol (HR =0.73 [0.5 to 1.1] and HR =1.26 [1.0 to 1.6] for women and men, respectively) concentrations, but the associations did not reach statistical significance (Figure 1A). In contrast to the cholesterol synthesis markers, cholesterol absorption markers were not predictive of HCHD in either women or men (Figure 1B). The cholesterol synthesis:absorption ratios (Figure 1C) tended to be lower in women and associated with higher risk in men, but only the lathosterol:sitosterol ratio reached significance in the women (HR=0.66 [0.4 to 0.9]). These ratios provide an overall assessment of cholesterol homeostasis because they take into account the relative contributions of cholesterol synthesis as well as absorption.^37^ We also assessed the prognostic value of the cholesterol homeostasis markers and full CVD (a composite of HCHD plus stroke, coronary insufficiency and angina pectoris, peripheral artery disease, and congestive heart failure). No significant associations were observed (Figure 2A through 2C); however, the sex‐specific trends observed for HCHD were similar to those observed with full CVD events, with the cholesterol synthesis markers being associated with lower risk in women and higher risk in men. Of note, the multivariable model adjusted for lipid parameters including LDL‐C. Similar results were obtained when LDL‐C was not included in the model. In addition, to determine if the cholesterol homeostasis markers could be used for risk prediction above and beyond LDL‐C, we calculated the net reclassification improvement after the addition of each marker into the model. Results suggest that there might be some improvement in risk classification for women when squalene was added to the model (0.1298 [−0.0188 to 0.5554]; net reclassification improvement with 95% CIs), but it was widely variable, possibility due to the smaller number of events in our cohort. No improvement was observed with the other cholesterol synthesis markers.

### Cholesterol Homeostasis Markers and CVD Risk Factors

In cross‐sectional analysis, marked differences on the basis of sex were observed in cholesterol homeostasis marker concentrations that were not explained by differences in BMI (Table 1). On average, women had significantly lower concentrations of all cholesterol absorption markers assessed compared with men (*P*<0.01). Among the cholesterol synthesis markers, squalene was significantly higher (*P*<0.01) in women than in men, whereas desmosterol (*P*<0.0001), but not lathosterol, was significantly lower in women. Regardless of these differences, the ratios of lathosterol to campesterol, sitosterol, and cholestanol were not significantly different between women and men.

**Table 1.** Plasma Noncholesterol Sterol Concentrations by Sex

Variable	Women (n=1463)	Men (n=1153)	*P* Value*	BMI Adjusted *P* Value*
Cholesterol synthesis markers,* 10^2^ mmol/mol of cholesterol				
Squalene	39.8±0.4	38.4±0.5	0.006	0.026
Desmosterol	53.8±0.6	59.4±0.8	<0.0001	<0.0001
Lathosterol	110.3±1.2	114.2±1.5	0.141	0.239
Cholesterol absorption markers,* 10^2^ mmol/mol of cholesterol				
Campesterol	214.8±2.5	225.0±2.9	0.002	<0.0001
Sitosterol	160.2±1.8	167.7±2.2	0.008	<0.0001
Cholestanol	120.7±1.2	126.2±1.4	0.002	0.004
Synthesis:absorption ratio				
Lathosterol:campesterol	0.64±0.01	0.61±0.01	0.177	**0.011**
Lathosterol:sitosterol	0.85±0.02	0.83±0.02	0.344	**0.021**
Lathosterol:cholestanol	1.03±0.01	1.03±0.02	0.290	0.239

* Values are mean±SE.

* *P* value based on 2‐sample *t* test.

* *P* value based on regression coefficient for sex from the model with body mass index (BMI).

In both women and men, older age was associated with higher desmosterol and lathosterol concentrations and lower squalene, campesterol, and sitosterol concentrations (Tables 2 and 3; correlations provided in Table 4). Women with higher BMI values (≥30 kg/m^2^) and waist circumferences (≥88 cm) had higher desmosterol and lathosterol concentrations and lower campesterol and sitosterol concentrations. Men with higher BMI values (≥30 kg/m^2^) and waist circumferences (≥102 cm) also had lower campesterol and sitosterol concentrations but, in contrast to women, without an apparent compensatory change in cholesterol synthesis marker concentrations.

**Table 2.** Plasma Cholesterol Homeostasis Marker Concentrations for Women by CVD Risk Factors

Variable	N	Cholesterol Synthesis Markers*			Cholesterol Absorption Markers*		
		Squalene	Desmosterol	Lathosterol	Campesterol	Sitosterol	Cholestanol
Age, y							
30 to 49	318	42.0±0.8	50.8±1.0	106.3±2.3	239.0±5.6	179.7±4.1	122.8±2.7
50 to 59	552	39.8±0.6	52.0±0.9	108.5±1.8	207.7±4.0	152.5±2.7	119.1±1.7
60 to 69	404	39.6±0.7	54.8±1.4	112.0±2.2	208.0±4.6	154.6±3.3	119.1±2.1
>70	189	36.1±1.1	62.4±2.3	118.4±3.9	209.5±7.9	162.2±5.8	125.1±3.8
*P* value*		<0.0001	<0.0001	0.022	<0.0001	<0.0001	0.682
Body mass index, kg/m^2^							
<25	603	40.5±0.6	51.2±0.8	106.0±1.6	240.4±4.3	181.5±3.1	119.2±1.9
≥25, <30	504	39.1±0.6	54.6±1.1	109.9±2.0	203.8±3.8	152.9±2.7	120.9±1.9
≥30	352	39.2±0.8	57.4±1.5	118.3±2.7	187.0±4.8	134.5±3.1	122.7±2.4
*P* value*		0.164	0.022	0.003	<0.0001	<0.0001	0.298
Waist circumference, cm							
≤88	592	40.4±0.6	50.8±0.8	104.4±1.5	240.2±4.2	181.9±3.1	120.8±1.9
>88	848	39.3±0.5	55.8±0.9	114.1±1.6	197.9±3.1	145.7±2.1	120.5±1.5
*P* value*		0.106	0.004	0.001	<0.0001	<0.0001	0.875
Smoking							
Nonsmoker	1243	39.9±0.4	53.9±0.7	110.2±1.2	213.3±2.8	160.5±2.0	120.9±1.3
Current smoker	220	39.0±1.0	53.5±1.7	110.9±3.1	223.7±5.9	159.0±4.4	119.5±3.2
*P* value*		0.362	0.914	0.931	0.037	0.919	0.425
Blood pressure (SBP/DBP), mm Hg							
<130/<85	797	41.4±0.5	52.6±0.8	109.3±1.5	224.1±3.5	167.8±2.4	121.6±1.6
≥130/≥85	666	37.7±0.5	55.2±1.0	111.5±1.8	203.7±3.7	151.2±2.7	119.5±1.7
*P* value*		<0.0001	0.257	0.495	<0.0001	<0.0001	0.272
Glucose, mg/dL							
<100	1017	40.1±0.4	52.1±0.7	108.8±1.3	225.5±3.1	169.8±2.2	120.1±1.4
≥100	426	38.8±0.7	58.3±1.5	114.3±2.4	188.9±4.2	137.0±2.9	122.7±2.1
*P* value*		0.011	0.002	0.086	<0.0001	<0.0001	0.237
LDL cholesterol,* mg/dL							
<100	331	42.8±0.8	55.2±1.3	114.9±2.4	218.3±5.5	164.7±4.1	138.0±2.7
≥100, <130	481	40.1±0.7	55.0±1.2	111.6±2.1	216.9±4.6	162.0±3.3	124.1±2.0
≥130, <160	399	39.0±0.7	53.2±1.3	109.5±2.3	210.5±4.8	157.7±3.3	115.6±2.1
≥160, <190	168	35.8±1.0	51.1±1.7	106.6±3.1	212.1±7.0	154.3±4.8	101.7±2.5
≥190	69	36.7±1.4	49.3±2.2	96.1±3.2	220.2±12.6	162.9±8.8	92.5±3.5
*P* value*		<0.0001	0.042	0.011	0.838	0.663	<0.0001
HDL cholesterol,* mg/dL							
≤40	168	40.5±1.2	54.6±1.9	108.4±3.7	191.0±7.0	140.0±4.8	127.3±3.5
40 to 60	648	39.3±0.5	54.3±1.0	109.7±1.7	208.8±3.8	157.0±2.7	120.1±1.8
≥60	644	40.0±0.6	53.2±0.9	111.4±1.7	227.1±3.9	168.9±2.8	119.6±1.7
*P* value*		0.713	0.818	0.245	<0.0001	<0.0001	0.086
Non–HDL cholesterol,* mg/dL							
<130	428	42.0±0.7	55.6±1.1	112.8±2.0	227.0±4.8	173.0±3.6	135.7±2.4
≥130, <160	459	39.4±0.6	53.8±1.2	111.9±2.2	212.6±4.4	158.0±3.1	120.6±2.0
≥160, <190	321	39.4±0.8	53.7±1.5	110.4±2.6	204.2±5.5	151.4±4.0	116.9±2.4
≥190	252	37.0±0.8	51.1±1.4	103.0±2.6	211.6±6.0	154.0±4.0	100.3±2.0
*P* value*		0.001	0.019	0.006	0.002	<0.0001	<0.0001
Triglycerides,* mg/dL							
<150	1059	40.0±0.4	54.1±0.7	110.6±1.3	225.8±3.1	169.8±2.2	122.2±1.4
≥150, <200	213	38.6±0.9	53.5±1.8	111.8±3.5	192.9±5.7	139.6±3.7	118.9±2.9
≥200	191	39.7±1.1	53.0±1.8	106.7±3.7	178.2±5.5	130.5±4.0	114.0±2.9
*P* value*		0.640	0.266	0.059	<0.0001	<0.0001	0.057
Metabolic syndrome							
Absence		40.3±0.4	52.2±0.7	108.8±1.3	228.9±3.2	172.7±2.3	120.6±1.4
Presence		38.5±0.7	57.2±1.3	113.7±2.3	186.8±3.7	135.6±2.6	120.8±2.0
*P* value*		0.02	0.0002	0.05	<0.0001	<0.0001	0.94
Diabetes mellitus							
Absence	1369	39.8±0.4	53.1±0.6	110.0±1.2	216.6±2.6	161.9±1.9	120.3±1.2
Presence	94	38.4±1.4	64.4±4.0	114.3±5.2	189.1±9.4	136.7±6.3	125.9±4.9
*P* value*		0.366	0.006	0.656	0.002	0.0002	0.261

CVD indicates cardiovascular disease; SBP, systolic blood pressure; DBP, diastolic blood pressure; LDL, low‐density lipoprotein; HDL, high‐density lipoprotein.

* Values are mean±SE.

* *P* value based on ANOVA.

* To convert values for cholesterol and triglycerides to millimoles per liter, divide by 38.67 and 88.54, respectively.

**Table 3.** Plasma Cholesterol Homeostasis Marker Concentrations for Men by CVD Risk Factors

Variable	N	Cholesterol Synthesis Markers*			Cholesterol Absorption Markers*		
		Squalene	Desmosterol	Lathosterol	Campesterol	Sitosterol	Cholestanol
Age, y							
30 to 49	245	40.2±0.8	55.0±1.3	109.5±2.9	258.2±7.6	187.4±5.4	123.0±2.7
50 to 59	450	38.8±0.6	55.5±1.2	108.8±2.1	225.3±4.6	165.7±3.3	125.6±2.1
60 to 69	322	38.1±1.0	61.4±1.6	119.6±3.0	215.1±5.0	163.4±3.8	130.0±2.6
>70	136	35.0±1.7	75.6±2.8	127.6±4.8	187.8±6.0	148.9±5.5	124.7±4.5
*P* value*		<0.0001	<0.0001	0.0004	<0.0001	<0.0001	0.299
Body mass index, kg/m^2^							
<25	251	38.2±0.8	61.7±1.7	113.9±2.9	239.0±6.1	182.6±4.5	126.8±2.9
≥25, <30	567	38.6±0.6	58.8±1.1	113.7±2.1	234.3±4.5	174.4±3.3	124.1±1.9
≥30	331	38.5±1.0	58.7±1.6	115.4±2.9	199.4±4.5	145.4±3.3	129.5±2.6
*P* value*		0.825	0.078	0.928	<0.0001	<0.0001	0.221
Waist circumference, cm							
≤102	668	38.2±0.5	59.6±1.0	112.6±1.8	240.1±4.1	179.6±3.0	125.6±1.8
>102	480	38.7±0.8	59.0±1.4	116.6±2.3	204.3±3.9	151.6±2.9	126.7±2.1
*P* value*		0.713	0.212	0.200	<0.0001	<0.0001	0.835
Smoking							
Nonsmoker	985	38.6±0.5	59.6±0.9	114.7±1.6	225.2±3.2	169.3±2.3	124.9±1.5
Current smoker	168	37.7±1.0	58.3±2.1	111.1±4.0	224.2±8.0	158.4±5.5	133.5±3.5
*P* value*		0.801	0.571	0.199	0.498	0.017	0.017
Blood pressure (SBP/DBP), mm Hg							
<130/<85	503	39.0±0.6	56.7±1.0	114.0±2.1	239.8±4.5	179.4±3.3	128.1±2.1
≥130/≥85	649	38.0±0.6	61.5±1.2	114.3±2.0	213.2±3.8	158.4±2.8	124.7±1.8
*P* value*		0.086	0.054	0.728	<0.0001	<0.0001	0.183
Glucose, mg/dL							
<100	565	38.6±0.6	60.3±1.2	115.0±2.0	235.4±4.3	176.4±3.2	128.0±1.9
≥100	555	38.2±0.7	58.6±1.2	114.1±2.2	214.3±4.1	159.0±2.9	124.4±2.0
*P* value*		0.338	0.280	0.485	<0.0001	<0.0001	0.086
LDL cholesterol,* mg/dL							
<100	186	41.1±1.4	68.6±2.1	121.7±3.9	223.3±7.1	166.1±5.4	146.5±4.0
≥100, <130	412	38.6±0.7	60.1±1.4	114.5±2.5	220.4±4.1	163.9±3.2	131.8±2.2
≥130, <160	341	38.2±0.7	56.8±1.4	115.0±2.6	231.7±5.9	173.9±4.2	118.9±2.3
≥160, <190	159	34.4±1.1	54.5±1.9	104.7±3.3	231.8±9.5	169.8±6.5	111.2±2.5
≥190	35	36.0±2.1	52.3±4.1	101.5±5.9	217.0±16.3	165.6±12.0	94.2±6.9
*P* value*		0.001	<0.0001	0.016	0.929	0.727	<0.0001
HDL cholesterol,* mg/dL							
≤40	476	40.1±0.8	60.0±1.4	117.9±2.5	210.5±3.8	156.8±2.9	129.2±2.2
40 to 60	541	37.5±0.6	58.6±1.1	111.4±2.0	235.0±4.7	173.8±3.3	125.2±1.9
≥60	136	36.3±1.1	60.6±2.0	112.0±3.6	236.2±8.8	181.5±7.0	119.7±3.9
*P* value*		0.007	0.448	0.392	0.001	0.0002	0.108
Non–HDL cholesterol,* mg/dL							
<130	263	39.3±1.0	64.5±1.7	115.8±2.9	231.0±5.8	172.8±4.5	143.2±3.3
≥130, <160	359	38.3±0.7	60.9±1.5	116.7±2.8	220.0±4.9	163.7±3.5	127.6±2.3
≥160, <190	335	38.7±0.9	57.9±1.6	115.0±2.8	229.5±5.9	170.2±4.4	123.3±2.3
≥190	196	37.0±1.3	52.4±1.8	105.9±3.0	218.6±7.3	163.8±5.2	105.6±2.6
*P* value*		0.284	<0.0001	0.112	0.227	0.377	<0.0001
Triglycerides,* mg/dL							
<150	797	37.6±0.5	59.5±0.9	110.0±1.5	235.0±3.7	174.3±2.7	127.2±1.6
≥150, <200	168	38.4±1.0	58.8±2.3	118.9±4.3	206.9±6.4	159.0±5.1	123.4±3.6
≥200	188	41.9±1.6	59.6±2.4	127.3±4.7	199.0±6.0	147.6±4.5	124.3±3.4
*P* value*		0.053	0.2306	0.003	<0.0001	<0.0001	0.337
Metabolic syndrome							
Absence	699	37.7±0.5	59.3±1.0	110.7±1.7	239.2±4.0	179.6±2.9	126.4±1.7
Presence	444	39.6±0.8	59.5±1.5	120.1±2.7	202.3±4.0	148.5±2.9	125.9±2.3
*P* value*		0.04	0.90	0.002	<0.0001	<0.0001	0.85
Diabetes mellitus							
Absence	1058	38.7±0.5	58.8±0.8	113.7±1.5	227.8±3.1	169.6±2.3	125.7±1.4
Presence	93	35.9±1.5	66.0±3.5	119.7±6.1	194.3±8.0	147.3±6.3	133.1±5.7
*P* value*		0.039	0.073	0.571	0.001	0.005	0.302

CVD indicates cardiovascular disease; SBP, systolic blood pressure; DBP, diastolic blood pressure; LDL, low‐density lipoprotein; HDL, high‐density lipoprotein.

* Values are mean±SE.

* *P* value based on ANOVA.

* To convert values for cholesterol and triglycerides to millimoles per liter, divide by 38.67 and 88.54, respectively.

**Table 4.** Correlation Between Cholesterol Homeostasis Markers and CVD Risk Factors*

Variable	Women						Men					
	Age	BMI	LDL‐C	HDL‐C	Non–HDL‐C	TG*	Age	BMI	LDL‐C	HDL‐C	Non–HDL‐C	TG
Cholesterol synthesis markers*												
Squalene	−0.14*	−0.06*	−0.13*	0.02	−0.12*	−0.04	−0.16*	0.03	−0.10*	−0.09*	−0.04	0.07*
Desmosterol	0.11*	0.06*	−0.07*	−0.03	−0.07*	−0.03	0.19*	0.06	−0.17*	0.02	−0.18*	−0.08*
Lathosterol	0.07*	0.08*	−0.08*	0.02	−0.08*	−0.04	0.11*	0.02	−0.10*	−0.05	−0.06*	0.07*
Cholesterol absorption markers*												
Campesterol	−0.12*	−0.23*	−0.01	0.14*	−0.09*	−0.26*	−0.21*	−0.15*	0.02	0.10*	−0.04	−0.19*
Sitosterol	−0.12*	−0.30*	−0.03	0.14*	−0.12*	−0.31*	−0.17*	−0.20*	0.03	0.11*	−0.04	−0.19*
Cholestanol	−0.02	0.03	−0.31*	−0.04	−0.30*	−0.10*	−0.01	0.01	−0.27*	−0.07*	−0.28*	−0.08*
Cholesterol synthesis:absorption ratio*												
Lathosterol:campesterol	0.13*	0.22*	−0.04	−0.09*	0.01	0.16*	0.22*	0.12*	−0.08*	−0.10*	−0.01	0.18*
Lathosterol:sitosterol	0.13*	0.26*	−0.02	−0.08*	0.04	0.19*	0.19*	0.15*	−0.09*	−0.11*	−0.01	0.18*
Lathosterol:cholestanol	0.07*	0.04	0.17*	0.04	0.15*	0.03	0.09*	0.003	0.11*	0.01	0.14*	0.11*

CVD indicates cardiovascular disease; BMI, body mass index; LDL‐C, LDL cholesterol; HDL‐C, HDL cholesterol; TG, triglycerides.

* Pearson product moment correlation coefficient.

* Triglycerides and cholesterol synthesis and absorption markers and ratios were log‐transformed during analysis to correct for their skewed distributions.

* ^‡^*P*<0.05; ^§^*P*<0.01; ^¶^*P*<0.001.

There was some indication that smoking status was associated with altered cholesterol absorption but not synthesis marker concentrations. Among smokers, women had higher campesterol concentrations, whereas men had lower sitosterol and higher cholestanol concentrations. Regardless of sex, participants with high blood pressure (≥130/≥85 mm Hg) and high glucose (≥100 mg/dL) concentrations had lower campesterol and sitosterol concentrations. However, women with high blood pressure and high glucose concentrations also had differences in the cholesterol synthesis marker concentrations characterized by lower squalene and higher desmosterol and a trend toward higher lathosterol concentrations. Such relationships or trends in relationships were not observed in the men.

With regard to plasma lipid profiles (Tables 2 and 3), lower cholesterol absorption (campesterol and sitosterol concentrations) was associated with higher triglyceride and lower HDL‐C concentrations, whereas lower cholesterol synthesis (squalene, desmosterol, and lathosterol concentrations) was associated with higher LDL‐C concentrations in both women and men. Sex differences were observed in non–HDL‐C and triglyceride concentrations. In women, lower cholesterol synthesis and absorption marker concentrations were associated with higher non–HDL‐C concentrations, whereas in men, higher lathosterol concentrations were associated with higher triglyceride concentrations.

Given the association between the cholesterol absorption/synthesis markers and several CVD risk factors that comprise or contribute to MetS and type 2 diabetes, we further assessed whether the presence or absence of these metabolic conditions was related to differences in cholesterol homeostasis (Tables 2 and 3). Women and men classified as having MetS or diabetes had lower campesterol and sitosterol concentrations (both *P*<0.05) and higher desmosterol and lathosterol concentrations (*P*<0.05 for desmosterol and lathosterol in women; *P*<0.05 for lathosterol in men). Squalene concentrations were lower in women with MetS (−4.5%, *P*=0.02) but higher in men with MetS (5%, *P*=0.04) compared with their counterparts not classified as having MetS. Similarly, diabetic women had higher desmosterol concentrations (21%, *P*<0.01), whereas diabetic men had lower squalene concentrations (−7%, *P*=0.04) with no difference in desmosterol or lathosterol concentrations.

## Discussion

In cross‐sectional analyses from this community‐based observational study, significant differences in cholesterol absorption and synthesis markers by sex, age, BMI, blood pressure, lipoprotein concentrations, smoking, and metabolic status were observed. When studied prospectively during a 10‐year period, after controlling for standard CVD risk factors, the cholesterol synthesis markers predicted HCHD; however, sex‐specific differences were observed. In women, squalene was associated with lower risk of coronary death and incidence of myocardial infarction, whereas in men, it was associated with higher risk of coronary death and incidence of myocardial infarction.

**Figure 3.**
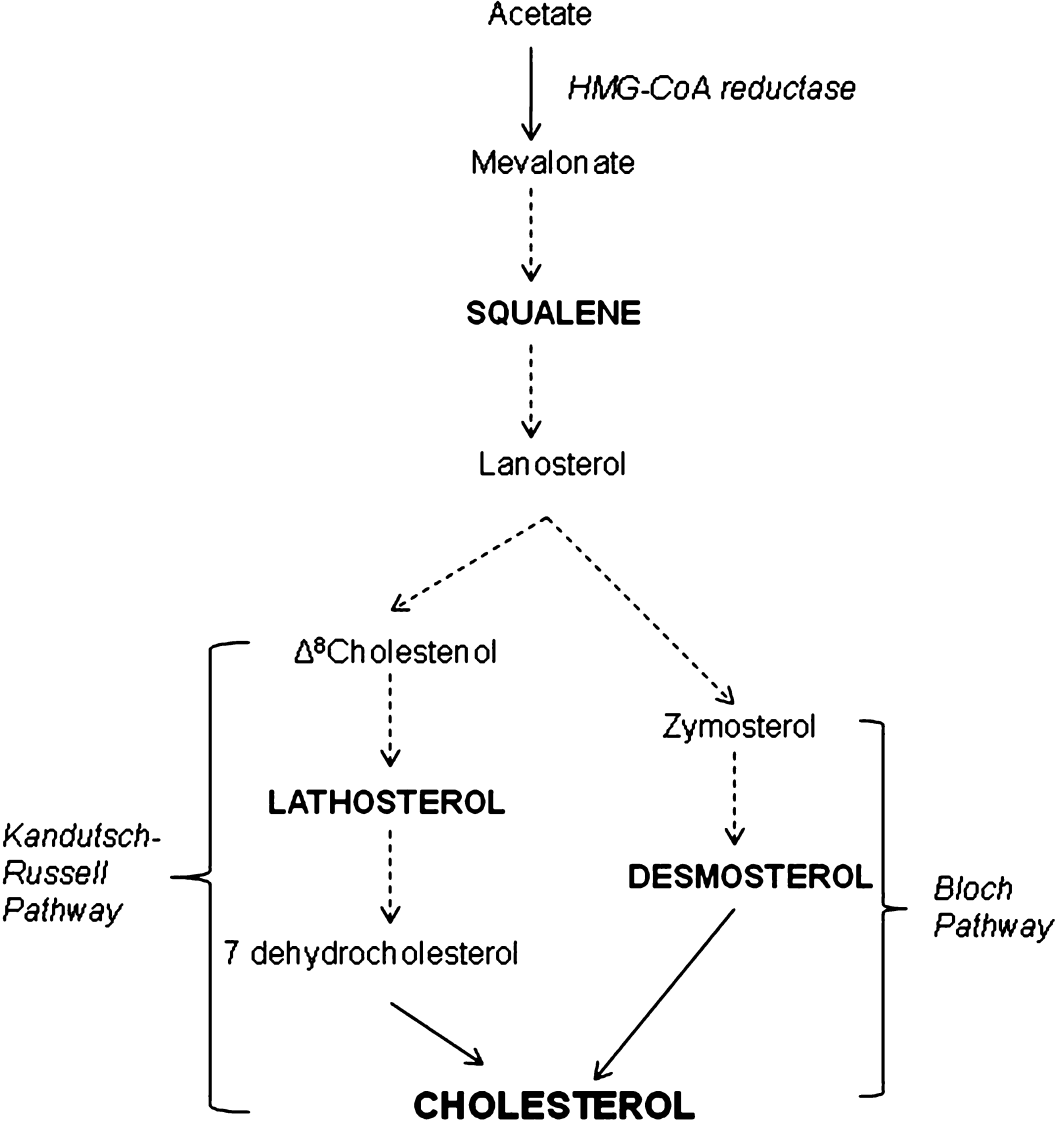
Cholesterol biosynthetic pathway highlighting the formation of squalene, an early intermediate, as well as desmosterol (Bloch pathway) and lathosterol (Kandutsch–Russell pathway) formed from squalene later in the biosynthetic pathway. HMG‐CoA indicates 3‐hydroxy‐3‐methylglutaryl–coenzyme A.

To our knowledge, this is the largest and most comprehensive study to date to examine the relationship between cholesterol homeostasis marker concentrations and CVD risk factors and events. To adequately address the primary and secondary aims of this work, we measured one cholesterol synthesis marker, squalene, formed as an early intermediate in the biosynthetic pathway, as well as 2 cholesterol synthesis markers formed from squalene later in the biosynthetic pathway, desmosterol and lathosterol, representing 2 alternate cholesterol biosynthetic pathways (Figure 3). In the Kandutsch–Russell pathway, lathosterol is a precursor of cholesterol, and in the Bloch pathway, desmosterol is a precursor of cholesterol. It is thought that these pathways may be independently regulated.^38^ Among the cholesterol absorption markers, we measured campesterol and sitosterol, which are derived from the diet, as well as cholestanol, a 5α saturated derivative of cholesterol, which is not influenced by dietary intake.

Several case–control studies have assessed the relationship between cholesterol homeostasis marker concentrations and prevalent CVD risk, but the results have been inconsistent.^18–30^ Data are more limited regarding the association between cholesterol homeostasis markers and CVD events. Among the 4 studies identified, the Drugs and Evidence BAsed medicine in The Elderly (DEBATE) study^39^ included 247 women and 149 men aged >75 years with documented CVD and abnormalities in glucose metabolism and reported that individuals in the highest quartile of cholestanol concentrations had the greatest increase in mortality (HR=3.53 [1.52 to 8.19]) and recurrence of CVD events relative to the lowest quartile. In addition, in this cohort, cholestanol and sitosterol, but not lathosterol, concentrations were higher in individuals who died after 3.4 years of follow‐up. However, given the age of the cohort, survivor bias cannot be ruled out. The LUdwigshafen RIsk and Cardiovascular health (LURIC)^20^ and the Helsinki Aging^40^ studies also focused on an elderly population. In the LURIC study, 1257 participants with CVD and not taking statins were followed for ≈7 years. All‐cause and CVD mortality rates were lower in individuals in the highest versus the lowest lathosterol tertile (HR=0.61 [0.5 to 0.8] and HR=0.60 [0.4 to 0.9) for all‐cause and CVD mortality, respectively), whereas the third cholestanol tertile was associated with increased all‐cause (HR=1.71 [1.3 to 2.3]) and CVD (HR=1.60 [1.1 to 2.3]) mortality. No significant association was observed across sitosterol tertiles. Similar results were observed in the Helsinki Businessmen Study ^41^, which followed 232 middle‐aged men with CVD and not using statin therapy for a period of 22 years. In multivariate analysis, men in the highest sitosterol tertile had a 42% to 47% lower mortality rate (HR=0.51 [0.3 to 0.9]) than those in the highest desmosterol to sitosterol tertile and had a 67% higher mortality rate (HR=1.67 [1.1 to 2.5]) than those men in the lowest tertiles. Taken together, these results suggest that higher cholesterol synthesis and lower absorption predict long‐term mortality. In contrast, low cholesterol synthesis and low cholesterol absorption were associated with higher mortality in 1 of the 2 studies.

Expanding the previous data sets available on cholesterol homeostasis markers and CVD risk factors and outcomes, our study identified notable sex‐specific associations between some of the cholesterol synthesis marker concentrations and HCHD. In women, squalene was associated with a 30% lower incidence of myocardial infarction and coronary death. Desmosterol (29%) and lathosterol (28%) showed similar but nonsignificant protective trends. In men, squalene was associated with 40% higher risk of coronary death and myocardial infarction. Desmosterol (19%) and lathosterol (26%) showed similar risk trends. Sex‐specific differences were not observed in the LURIC study; however, given the age of the cohort, it might have been difficult to tease out these differences. The other 2 studies were not powered to determine sex‐specific associations. We did not find an association between cholesterol absorption markers and HCHD or full CVD.

In our cross‐sectional analysis, women had lower concentrations of the absorption and synthesis markers compared with men, and this was not explained by differences in BMI. This is consistent with some,^26–27,42^ but not all, of the prior data available.^25,20,43^ As indicated previously, limited sample size in several of the prior studies may have precluded analysis on the basis of sex. A novel finding of the present work is that squalene concentrations were markedly higher in women relative to men. Squalene can be obtained in small amounts from foods (eg, olive oil), but because this was not a predominant component of the diet, the higher concentrations in women most likely reflect lower conversion to cholesterol via the sterol pathways than in men. Were this hypothesis correct, it could also account for the lower desmosterol and lathosterol concentrations observed in men compared with women in our cohort.

Increasing age was associated with lower squalene and higher desmosterol and lathosterol concentrations, a finding observed in both sexes. Consistent with prior reports, we also observed that higher cholesterol synthesis marker concentrations were associated with lower absorption marker concentrations.^19–20,40,42^ Changes in body composition (loss of lean protein mass and increase in fat mass) could partially account for these age‐related changes.^44^ This hypothesis is supported by our findings of lower cholesterol absorption marker concentrations in men and women and of higher synthesis marker concentrations in women with higher BMI values and waist circumferences, as well prior observations in obese subjects^17,45–47^ and those with MetS^48–49^ and diabetes.^50–53^

Lower cholesterol absorption marker concentrations were associated with higher triglyceride and lower HDL‐C concentrations, whereas lower cholesterol synthesis marker concentrations were associated with higher LDL‐C concentrations, in both women and men. These results are consistent with previous reports that have had the power to address sex differences.^22,26,20^ The inverse association between cholesterol synthesis marker concentrations and plasma LDL‐C concentrations has been attributed to upregulation of the LDL receptor, which, in turn, increases internalization of LDL from the circulation leading to release of cholesterol into the cell, thereby downregulating HMG‐CoA reductase activity, the rate‐limiting enzyme in cholesterol biosynthesis.^54^

The association between non–HDL‐C concentrations and cholesterol homeostasis marker concentrations was also assessed in the present study given the strong association between non–HDL‐C and the pathophysiology of atherosclerosis^55^ and the documented sex‐specific difference in non–HDL‐C concentrations between women and men.^56^ Our results indicate that the lower non–HDL‐C concentrations in women are associated with lower cholesterol synthesis and absorption.

Although the regulatory mechanisms of cholesterol homeostasis have been established, little data are available to speculate on the mechanism(s) responsible for the sex‐specific differences observed. HMG‐CoA reductase activity, the rate‐limiting enzyme in cholesterol biosynthesis, has been reported to be is lower in women than in men.^57^ It has been suggested that this factor contributes, in part, to a lower lifetime burden of cholesterol in women^41^ and, thus, lower event rates. Consistent with these data, in our cohort, squalene and, to a lesser extent, desmosterol and lathosterol were associated with a lower risk of HCHD in women but not in men. These markers are intermediates of cholesterol biosynthesis that have been correlated with HMG‐CoA reductase activity.^58^

A limitation of this study is that the associations observed could have been influenced by factors that we were not able to control for, such as genotype. Second, the phytosterol content of the diets could not be assessed. However, plasma samples were collected before the introduction of phytosterol‐enriched products into the marketplace, which would eliminated this potential source of confounding. Third, only a single determination of cholesterol absorption and synthesis markers was made during cycle 6, which could have been responsible for some of the variability in the data. To minimize this variation, only fasted samples collected according to a strict protocol within a specified timeframe were used. Finally, the FOS population studied was predominantly white, so care must be taken in extrapolating the results to other populations.

In conclusion, our results suggest significant sex differences in the 10‐year prognostic value of cholesterol synthesis markers, specifically squalene, on coronary death and incidence of myocardial infarction (HCHD). This could have implications in the management of CVD risk and choice of lipid‐lowering therapy, including newer agents such as the squalene synthase inhibitors.
